# Comparison of endovascular and microsurgical treatment in patients with aneurysmal subarachnoid hemorrhage requiring external ventricular drainage

**DOI:** 10.3389/fneur.2025.1708743

**Published:** 2026-01-26

**Authors:** Xinwang Cai, Xiuhu An, Kaijie Wang, Jianqiang Wei, Yang Guo, Bangyue Wang, Yan Zhao, Xinyu Yang, Jianzhong Cui

**Affiliations:** 1 Department of Surgery, Hebei Medical University, Shijiazhuang, China; 2 Department of Neurosurgery, Tangshan Gongren Hospital, Tangshan, China; 3 Department of Neurosurgery, Tianjin Medical University General Hospital, Tianjin, China; 4 Department of Anesthesiology, Tianjin Medical University General Hospital, Tianjin, China; 5 Department of Neurosurgery, The Second Affiliated Hospital of Anhui Medical University, Hefei, China

**Keywords:** aneurysmal subarachnoid hemorrhage, endovascular treatment, external ventricular drainage, microsurgical treatment, outcome

## Abstract

**Background:**

The efficacy of microsurgical treatment (MST) and endovascular treatment (EVT) in aneurysmal subarachnoid hemorrhage (aSAH) patients requiring external ventricular drainage (EVD) remains unclear. This study aims to comprehensively compare the outcomes of MST and EVT in this specific patient population.

**Methods:**

We consecutively enrolled surgical patients with aSAH requiring EVD from the Chinese Multicenter Aneurysm Database (CMAD) between January 2017 and December 2020. A 1:1 propensity score matching (PSM) was performed to balance baseline differences between the MST and EVT groups. Outcomes and complications were then compared between the matched groups. Logistic regression was used to calculate odds ratios (ORs) and 95% confidence intervals (CIs). The Kaplan–Meier survival curves were used to compare survival between the MST and EVT groups.

**Results:**

A total of 197 aSAH patients met the inclusion criteria. After PSM, 45 patients who underwent MST were matched with 45 patients who received EVT. No significant differences were observed between the MST and EVT groups in terms of 2-year mortality (MST: 32.3%; EVT: 35.5%, *p* = 0.48), dependent survival at discharge (MST: 51.2%; EVT: 48.8%, OR 0.955, 95% CI 0.399–2.285, *p* = 0.917), or dependent survival at 2 years (MST: 70.8%; EVT: 29.2%, OR 1.080, 95% CI 0.253–4.607, *p* = 0.918). Compared with the EVT group, the MST group had a significantly higher incidence of intracranial infection (MST: 26.7%; EVT: 4.4%, OR 0.128, 95% CI 0.027–0.611, *p* = 0.010) and a lower incidence of pneumonia (MST: 22.2%; EVT: 42.2%, OR 2.558, 95% CI 1.021–6.409, *p* = 0.045).

**Conclusion:**

In aSAH patients requiring EVD, EVT did not demonstrate clear advantages over MST in terms of survival or functional outcomes. MST was associated with a higher incidence of intracranial infection, whereas EVT showed a relatively higher rate of pneumonia during hospitalization. Given the retrospective design and limited sample size, these findings should be interpreted with caution.

## Introduction

Subarachnoid hemorrhage (SAH) is a life-threatening neurological emergency characterized by high rates of mortality and disability (1, 2). Approximately 85% of SAH cases are caused by the rupture of intracranial aneurysms (3). Microsurgical treatment (MST) and endovascular treatment (EVT) are the primary approaches for preventing aneurysmal rebleeding and have been shown to significantly reduce mortality (4). External ventricular drainage (EVD) serves as a life-saving intervention in aneurysmal SAH (aSAH) patients who develop acute hydrocephalus or elevated intracranial pressure. EVD can effectively reduce intracranial pressure and clear blood from the subarachnoid space, thereby lowering the risk of complications and improving prognosis (5).

Numerous studies have aimed to compare the outcomes of MST and EVT in treating aSAH. Notably, the two most prominent trials—the International Subarachnoid Aneurysm Trial (ISAT) and the Barrow Ruptured Aneurysm Trial (BRAT)—have indicated that EVT is associated with more favorable prognoses compared to MST (6–10). Since these randomized controlled trials (RCTs), EVT has become the preferred treatment strategy for aSAH. However, over the past decade, new adjunctive coiling devices such as balloons and stents have been introduced. Compared with conventional coil embolization, the use of these devices has been linked to an increased risk of treatment-related complications (11, 12). Moreover, aSAH patients requiring EVD tend to present with more severe disease, and fluctuations in intracranial pressure are closely associated with the risk of aneurysmal rebleeding (13). MST may offer advantages in such cases, including more effective evacuation of intracranial hematomas, decompressive craniectomy, and rapid management of rebleeding. These factors make treatment selection in this population particularly challenging. To date, no studies have specifically investigated the comparative effectiveness of MST and EVT in aSAH patients requiring EVD. Therefore, we conducted a multicenter follow-up study to comprehensively compare the outcomes of MST and EVT in this high-risk subgroup.

## Methods

### Study design

The Chinese Multicenter Aneurysm Database (CMAD) is a registered, multicenter, retrospective observational database in China (Clinical trial number: not applicable). This database consecutively enrolled patients clinically diagnosed with aSAH from 12 neurosurgical centers across China between January 2017 and December 2020. The study protocol was approved by the Ethics Committee of Tianjin Medical University General Hospital (IRB2022-YX-175-01). As only routinely collected clinical data were recorded, the requirement for informed consent was waived. All analyses were conducted in accordance with the Declaration of Helsinki and local ethical guidelines. Written informed consent for clinical procedures, including aneurysm surgery, was obtained from patients or their legal representatives prior to treatment. Surgical treatments were performed by neurosurgeons at reputable medical centers, with specific treatment details left to the discretion of the surgeons. For all patients undergoing EVD, the procedure was performed with informed consent obtained from family members based on clinical necessity. All patients were managed in accordance with the guidelines of the American Heart Association/American Stroke Association and the Chinese Expert Consensus on Neurosurgical Cerebrospinal Fluid External Drainage (2018 edition) (14, 15).

### Population

The inclusion criteria for this study were as follows: (1) age ≥18 years, (2) diagnosis of SAH confirmed by head CT or lumbar puncture; (3) diagnosis of saccular intracranial aneurysm confirmed by at least one imaging modality: digital subtraction angiography (DSA), computed tomography angiography (CTA), or magnetic resonance angiography (MRA); (4) underwent surgical treatment for the aneurysm and received EVD; and (5) aneurysm treatment performed within 48 h of SAH onset.

The exclusion criteria for this study were as follows: (1) presence of fungal, dissecting, fusiform, or traumatic aneurysms; (2) concurrent arteriovenous malformations, arteriovenous fistulas, or moyamoya disease; (3) severe comorbidities such as cardiopulmonary insufficiency; (4) patients who received both MST and EVT; and (5) patients who underwent lumbar drainage (LD).

### Data collection

Patient demographic and clinical data were collected from medical records (both paper and electronic), including age, sex, residence, history of hypertension, diabetes mellitus, prior stroke, smoking history, alcohol consumption, time from symptom onset to surgery, presence of intracerebral hemorrhage (ICH) or intraventricular hemorrhage (IVH), Hunt-Hess (HH) grade, World Federation of Neurosurgical Societies (WFNS) grade, location and size of the responsible aneurysm, number of aneurysms, and preoperative hydrocephalus. Surgical methods included MST (clipping, clipping with bypass) and EVT (coiling, stent placement, and flow diversion). Postoperative clinical complications during hospitalization were recorded, including cerebral ischemia, rebleeding, intracranial infection, pneumonia, stress ulcer bleeding, deep vein thrombosis (DVT), and postoperative hydrocephalus. At discharge, length of stay (LOS), survival status, and modified Rankin Scale (mRS) scores (0: no symptoms; 6: death) were collected.

### Outcome assessment

Outcome measures were assessed by trained research interviewers via telephone follow-up. The primary endpoint was all-cause mortality. The secondary endpoint was functional outcome at 2-year post-aSAH, evaluated using the mRS, with functional independence defined as mRS scores of 0–2 and dependency defined as mRS scores of 3–5.

### Statistical analysis

For descriptive analysis, frequencies (percentages) were used for categorical variables, while means (standard deviations, SD) or medians (interquartile ranges, IQR) were used for continuous variables. Pearson’s chi-squared test, continuity correction test, or Fisher’s exact test were applied to compare categorical variables, and the non-parametric Mann–Whitney *U*-test was used to compare continuous variables.

To compare outcomes and in-hospital complications between groups, propensity score matching (PSM) was performed to adjust for baseline differences. The variables included in the matching were age, sex, residence, hypertension, diabetes mellitus, prior stroke, smoking history, alcohol consumption, time from symptom onset to surgery, presence of ICH/IVH, HH grade, WFNS grade, location and size of the responsible aneurysm, number of aneurysms, and preoperative hydrocephalus. Nearest neighbor matching without replacement was used, with a caliper of 0.02 and at a 1:1 matching ratio. Logistic regression analysis was used to calculate odds ratios (ORs) and 95% confidence intervals (CIs). The Kaplan–Meier survival curves were used to compare survival between the MST and EVT groups.

All statistical analyses were performed using SPSS version 27 (IBM Corp., Armonk, NY, USA) and R version 4.4.1 (R Foundation for Statistical Computing, Vienna, Austria). Statistical significance was set at *p* < 0.05, and all confidence intervals are presented at 95% level.

### Bias mitigation

To reduce potential sources of bias inherent in this retrospective study, several methodological strategies were implemented:.

(1) To minimize potential sources of bias inherent to the retrospective design, several methodological strategies were applied. Consecutive patients meeting predefined inclusion and exclusion criteria were enrolled within a fixed study period, and restriction to patients requiring EVD was used to improve cohort homogeneity and reduce selection bias; (2) data were collected using standardized procedures. Treatment allocation to EVT or MST was based on multidisciplinary clinical decision-making rather than study-related considerations; and (3) PSM was performed to adjust for baseline imbalances and potential confounding, with uniform outcome definitions applied across groups. To assess whether missing data influenced the results, baseline characteristics were compared between patients who completed follow-up and those who lost follow-up.

## Results

**Figure 1:**
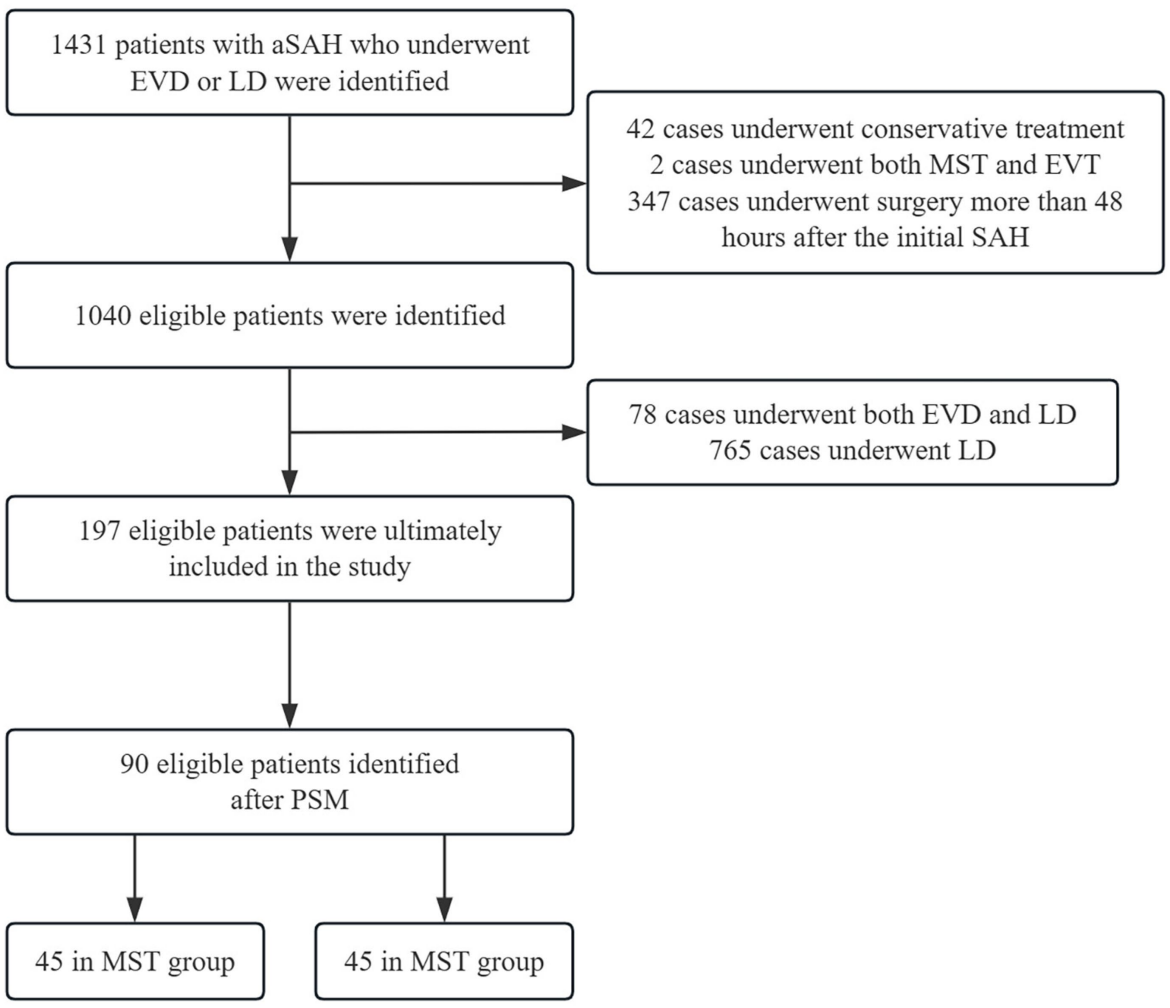
Detailed enrollment flowchart. SAH, Subarachnoid hemorrhage; EVD, external ventricular drainage; LD, lumbar drainage; MST, microsurgical treatment; EVT, endovascular treatment; PSM, propensity score matching.

Between January 2017 and December 2020, 1,431 patients who underwent cerebrospinal fluid drainage were initially screened. After applying the predefined inclusion and exclusion criteria, a total of 197 patients were included in the final analysis. Specifically, 42 patients received conservative treatment, 2 patients underwent both MST and EVT, 347 patients had aneurysm surgery performed more than 48 h after symptom onset, 78 patients received both EVD and LD, and 765 patients received LD only. The detailed enrollment flowchart is shown in Figure 1. Compared to the EVT group, the MST group had a longer time from symptom onset to surgery (MST: 21 [13–31] h; EVT: 18 [8–26] h, *p* = 0.011), and lower proportions of patients with HH grades IV–V (MST: 34.3%; EVT: 56.2%, *p* = 0.002) and WFNS grades IV–V (MST: 41.7%; EVT: 69.7%, *p* < 0.001). The MST group also had a lower proportion of aneurysms located in the posterior circulation (MST: 0.9%; EVT: 25.8%, *p* < 0.001) and a lower rate of pre-drainage hydrocephalus (MST: 1.9%; EVT: 18.0%, p < 0.001). After propensity score matching, 45 patients in the MST group were matched with 45 patients in the EVT group. No significant differences in baseline characteristics were observed after matching. (Table 1).

**Table 1:** Baseline characteristics before and after PSM.

Characteristics	Before PSM			After PSM		
	MST	EVT	*P* Value	MST	EVT	*p*-value
No. of patients	108	89		45	45	
Age, y; mean (SD)	58.6 (10.4)	61.0 (13.7)		57.5 (11.0)	60.9 (12.6)	
<65	74 (68.5)	49 (55.1)	0.052	30 (66.7)	27 (60.0)	0.512
≥65	34 (31.5)	40 (44.9)		15 (33.3)	18 (40.0)	
Sex, *n* (%)						0.827
Male	37 (34.3)	31 (34.8)	0.933	17 (37.8)	16 (35.6)	
Female	71 (65.7)	58 (65.2)		28 (62.2)	29 (64.4)	
Residence area, *n* (%)			0.507			0.624
Rural	81 (75.0)	63 (70.8)		35 (77.8)	33 (73.3)	
Urban	27 (25.0)	26 (39.2)		10 (22.2)	12 (26.7)	
Medical history, *n* (%)						
Hypertension	71 (65.7)	60 (67.4)	0.804	29 (64.4)	28 (62.2)	0.827
Diabetes	4 (3.7)	9 (10.1)	0.071	1 (2.2)	2 (4.4)	1
Previous stroke	19 (17.6)	13 (14.6)	0.572	5 (11.1)	6 (13.3)	0.748
Lifestyle risk factors, *n* (%)						
Smoking	18 (16.7)	12 (13.5)	0.536	7 (15.6)	4 (8.9)	0.334
Alcohol consumption	12 (11.1)	8 (10.1)	0.821	5 (11.1)	3 (6.7)	0.711
From onset to surgery, h; median (IQR)	21 (13–31)	18 (8–26)	0.011	17 (8–24)	23 (11–31)	0.159
Presence of ICH or IVH, *n* (%)	36 (33.3)	36 (40.4)	0.302	16 (35.6)	19 (42.2)	0.517
HH grade, *n* (%)			0.002			0.527
I-III	71 (65.7)	39 (43.8)		20 (44.4)	23 (51.1)	
IV-V	37 (34.3)	50 (56.2)		25 (55.6)	22 (48.9)	
WFNS grade, *n* (%)			<0.001			1
I-III	63 (58.3)	27 (30.3)		16 (35.6)	16 (35.6)	
IV-V	45 (41.7)	62 (69.7)		29 (64.4)	29 (64.4)	
Location of responsible aneurysm, *n* (%)			<0.001			1
Anterior circulation arteries	107 (99.1)	66 (74.2)		44 (97.8)	44 (97.8)	
Posterior circulation arteries	1 (0.9)	23 (25.8)		1 (2.2)	1 (2.2)	
Size of the responsible aneurysm, mm			0.736			0.673
<5	56 (51.9)	44 (49.4)		20 (44.4)	22 (48.9)	
≥5	52 (48.1)	45 (50.6)		25 (55.6)	23 (51.1)	
Multiple aneurysms, *n* (%)	14 (13.0)	20 (22.5)	0.079	8 (17.8)	5 (11.1)	0.368
Hydrocephalus before drainage, *n* (%)	2 (1.9)	16 (18.0)	<0.001	2 (4.4)	2 (4.4)	1

PSM, propensity score matching; MST, microsurgical treatment; EVT, endovascular treatment; ICH, intracranial hemorrhage; IVH, intraventricular hemorrhage; HH, Hunt-Hess grade.

In this cohort, the most common complications were pneumonia (32.2%) and intracranial infection (15.6%). Compared to the EVT group, the MST group had a higher incidence of intracranial infection (MST: 26.7%; EVT: 4.4%; OR 0.128, 95% CI 0.027–0.611, *p* = 0.010) but a lower incidence of pneumonia (MST: 22.2%; EVT: 42.2%; OR 2.558, 95% CI 1.021–6.409, *p* = 0.045). However, no significant differences were observed in discharge outcomes. (Table 2).

**Table 2:** Discharge characteristics and incidence of in-hospital complications in the MST versus EVT groups after PSM.

Variable	All patients (*n* = 90)	MST (*n* = 45)	EVT (*n* = 45)	OR (95%CI)	*p*-value
LOS, d, median (IQR)	21 (8–30)	19 (9–26)	24 (5–34)	NA	0.809
Mortality at discharge, *n* (%)	9 (10.0)	4 (8.9)	5 (11.1)	1.281 (0.321–5.119)	0.726
mRS score 3–6 at discharge, *n* (%)	52 (57.8)	26 (57.8)	26 (57.8)	1.000 (0.433–2.308)	1
In-hospital complications					
Cerebral ischemia	7 (7.8)	4 (8.9)	3 (6.7)	0.732 (0.154–3.476)	0.695
Rebleeding	9 (10.0)	4 (8.9)	5 (11.1)	1.281 (0.321–5.119)	0.726
Intracranial infection	14 (15.6)	12 (26.7)	2 (4.4)	0.128 (0.027–0.611)	0.010
Pneumonia	29 (32.2)	10 (22.2)	19 (42.2)	2.558 (1.021–6.409)	0.045
Stress ulcer bleeding	6 (6.7)	3 (6.7)	3 (6.7)	1.000 (0.191–5.241)	1
DVT	3 (3.3)	2 (4.4)	1 (2.2)	0.489 (0.043–5.589)	0.565
Hydrocephalus after drainage	2 (2.2)	0	2 (2.2)	NS	0.998

LOS, length of stay; NA, not applicable; DVT, deep vein thrombosis; NS, not significant.

**Figure 2:**
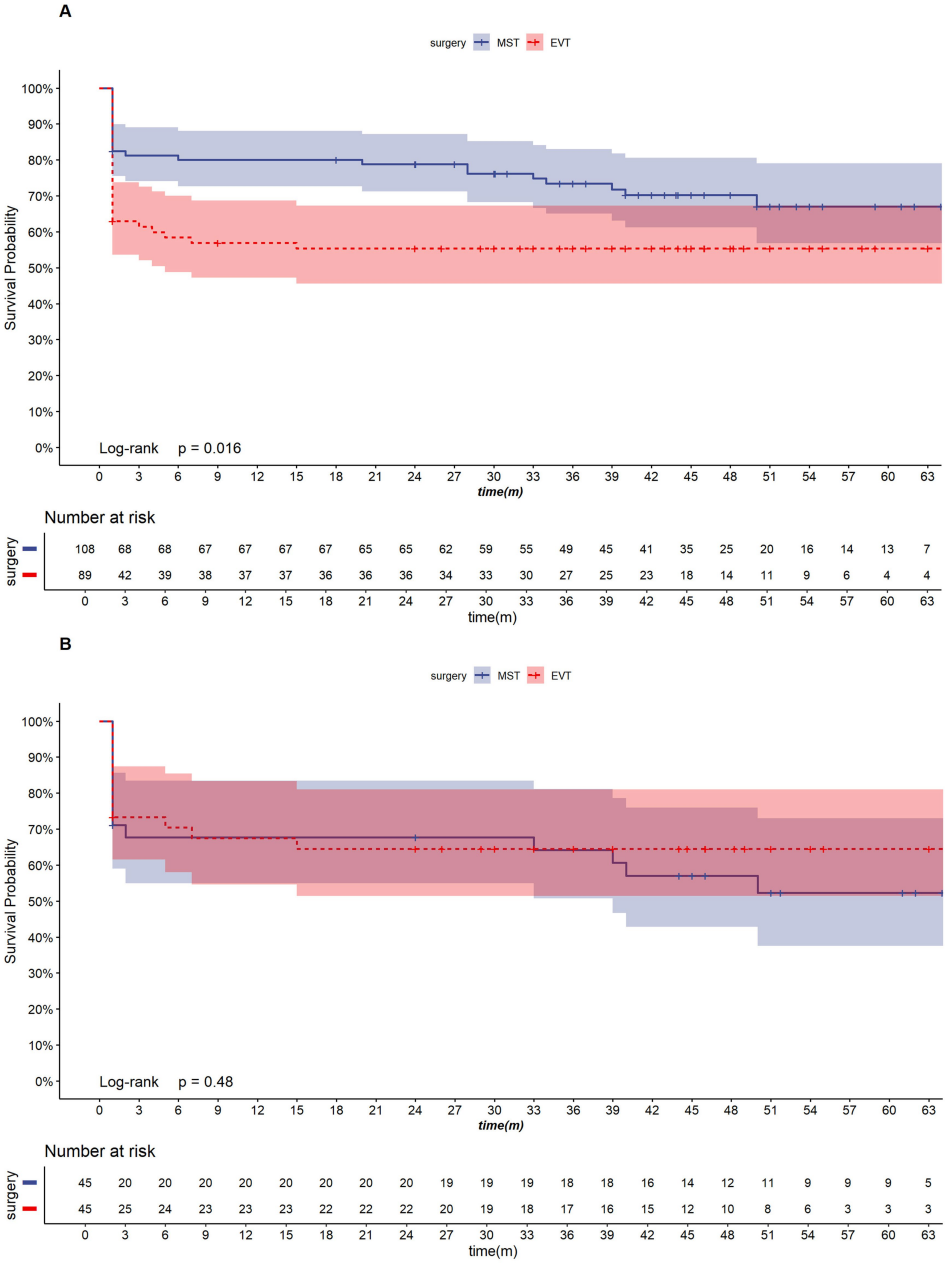
Kaplan–Meier survival curves for the MST and EVT groups. **(A)**: Pre-PSM; **(B)**: Post-PSM.

Figure 2 shows the Kaplan–Meier survival curves for the MST and EVT groups. A total of 197 patients were included in the survival analysis, with a mean follow-up duration of 24.2 ± 23.6 months. The overall mortality rates at 3 months and 1 year were 27.7 and 31.7%, respectively. Before propensity score matching (PSM), the EVT group had a higher 2-year mortality rate compared to the MST group (MST: 21.2%; EVT: 44.6%, *p* = 0.016). After PSM, there was no significant difference in 2-year mortality between the two groups (MST: 32.3%; EVT: 35.5%, *p* = 0.48) (Table 3).

**Table 3:** Comparison of 2-year cumulative mortality between MST and EVT.

Group	3 months (95%CI)	6 months (95%CI)	1 year (95%CI)	2 years (95%CI)
Before PSM				
Total	27.7% (21.1–33.8%)	29.7% (22.9–35.9%)	30.4% (23.5–36.7%)	31.7% (24.7–38.1%)
MST	18.8% (11.0–25.9%)	20.0% (11.9–27.3%)	20.0% (11.9–27.3%)	21.2% (12.8–28.7%)
EVT	38.6% (27.5–48.0%)	41.6% (30.0–51.2%)	43.1% (31.3–52.8%)	44.6% (32.7–54.4%)
After PSM				
Total	29.3% (19.1–38.3%)	30.9% (20.4–40.1%)	32.5% (21.6–41.8%)	34.1% (22.9–43.6%)
MST	32.3% (16.5–45.1%)	32.3% (16.5–45.1%)	32.3% (16.5–45.1%)	32.3% (16.5–45.1%)
EVT	26.7% (12.5–38.5%)	29.6% (14.6–42.0%)	32.5% (16.7–45.4%)	35.5% (18.9–48.6%)

A total of 55 patients survived and completed a 2-year follow-up. There were no significant differences between the MST and EVT groups in the proportion of dependent survival at discharge (MST: 51.2%; EVT: 48.8%; OR 0.955, 95% CI 0.399–2.285, *p* = 0.917) or at 2 years (MST: 70.8%; EVT: 29.2%; OR 1.080, 95% CI 0.253–4.607, *p* = 0.918) (Table 4). The detailed mRS distribution is shown in Supplementary Figure S1.

**Table 4:** Comparison of functional outcomes between MST and EVT.

Group	Dependent survival	Independent survival	OR (95%CI)	*p*-value
At discharge				
MST	22 (51.2)	19 (50.0)	Reference	
EVT	21 (48.8)	19 (50.0)	0.955 (0.399–2.285)	0.917
2 years				
MST	17 (70.8)	24 (92.3)	Reference	
EVT	7 (29.2)	2 (7.7)	1.080 (0.253–4.607)	0.918

## Discussion

Our findings indicate that, among aSAH patients requiring EVD, there were no significant differences between MST and EVT in terms of survival outcomes, functional outcomes, or LOS. Regarding in-hospital complications, the MST group showed a higher incidence of intracranial infection but a lower incidence of pneumonia compared to the EVT group. Our study utilized data from a large, multicenter, observational database with strict inclusion criteria, allowing for a more robust comparison of the efficacy of MST and EVT in treating aSAH. These results provide important evidence to inform clinical decision-making.

Numerous studies have investigated aSAH, but the majority of studies focus on the general population, with reported in-hospital mortality rates ranging from 10.9 to 27.5% (16–18). Galea et al. (19) reported a 3-month mortality rate of 30.7% in the general aSAH population. Consistent with existing evidence, the 3-month and 2-year mortality rates in our cohort of aSAH patients requiring EVD were 27.7 and 31.7%, respectively. The ISAT trial demonstrated significantly lower 5- and 10-year mortality risks after EVT compared to MST (20, 21). However, the ISAT results are not directly generalizable to all aneurysms, as the study primarily enrolled patients with good-grade anterior circulation aneurysms. Li et al. (22) also found that EVT had advantages over MST in discharge and 90-day outcomes, as well as in-hospital complications and the number of risk factors. Nevertheless, we observed a lower mortality rate in the MST group than in the EVT group, although the difference was not statistically significant after balancing baseline characteristics. This may be attributed to the presence of intracerebral hematoma, hemodynamic changes, hydrocephalus, and increased intracranial pressure in aSAH patients requiring EVD (23). MST not only treats the aneurysm but also allows hematoma evacuation, decompressive craniectomy, and rapid management of rebleeding. Additionally, EVD placement is generally more direct and convenient in the MST group compared to the EVT group. These advantages may balance the disadvantages associated with the greater invasiveness of MST.

Previous studies have shown that the placement of EVD is associated with improved clinical outcomes in aSAH patients (24–27). However, Gerner et al. (28) reported that the proportion of EVD placement was higher in non-survivors than in survivors, and the need for ventriculoperitoneal shunting was associated with a lower likelihood of achieving good functional outcomes, reduced chances of returning to work, and poorer self-reported health status. Therefore, in addition to analyzing survival outcomes, we also compared functional outcomes in aSAH patients requiring EVD. Although the majority of studies and reviews have reported that EVT is superior to MST in improving rates of favorable outcomes (20, 21, 29, 30), there was no significant difference in the proportion of dependent survival between the MST and EVT groups in the EVD-requiring aSAH population. This finding is consistent with ISAT results, which showed comparable rates of dependency between MST and EVT groups in the long term (31, 32). This may be related to the more severe condition and complex complications observed in this subgroup of aSAH patients requiring EVD.

EVD is associated with numerous complications, including intracranial hemorrhage, overdrainage and underdrainage syndromes, as well as infections (33, 34). Therefore, we compared the complications of MST and EVT in this population. EVD-related intracranial infection, including ventriculitis, is a well-recognized complication, with reported incidences ranging from 0 to 22% (35–39). Consistent with existing evidence, the intracranial infection rate in our study was 14.4%. Moreover, patients undergoing MST had a higher rate of intracranial infection than those receiving EVT. This may be due to the involvement of the central nervous system during craniotomy, the complexity and longer duration of surgery, and the blood–brain barrier limiting the effectiveness of antibiotics (40). EVD increases hospital and ICU stay and is often associated with prolonged mechanical ventilation, raising the risk of pneumonia (41). We observed a higher incidence of pneumonia in the EVT group, which may be related to the fact that 35.6% of EVD patients had ICH or IVH. In contrast, MST can simultaneously occlude the aneurysm and evacuate hematomas, thereby promoting earlier recovery of consciousness and airway protection. Although mechanical ventilation duration is an important risk factor, it was not consistently recorded. All patients were managed according to standardized protocols, which likely minimized differences in ventilation strategies. These mechanisms remain speculative and require further study. Furthermore, EVD use may influence the risk of other complications. Reduction of intracranial pressure can increase clot displacement, potentially raising the risk of rebleeding before aneurysm repair (13, 42, 43). Meta-analysis has reported a higher risk of rebleeding (18.8%) in aSAH patients requiring EVD (44). However, in our study, the overall rebleeding rate was lower (10.0%), possibly because some EVD placements occurred after aneurysm surgery, highlighting the importance of early aneurysm repair. Although the CARAT study (45) and ISAT (21) emphasized that EVT carries a higher risk of rebleeding compared to MST over longer follow-up periods, our study found similar efficacy of MST and EVT in preventing rebleeding in this population. These findings are consistent with those of Li et al. (22) Longer-term follow-up is still needed to further evaluate efficacy.

### Limitations

First, this study is retrospective in nature. Although consecutive enrollment and propensity score matching were used to balance baseline characteristics between the MST and EVT groups, residual confounding cannot be completely excluded. In addition, procedural heterogeneity within the EVT group, including the use of stent-assisted techniques, may have introduced further unmeasured confounding and should be considered when interpreting the results. Second, despite efforts to minimize loss to follow-up, 18.8% of patients were lost. Finally, the sample size was relatively small, and larger prospective studies are needed to further clarify these findings.

## Conclusion

In aSAH patients requiring EVD, EVT did not show clear advantages over MST in survival or long-term functional outcomes. MST was associated with a higher rate of intracranial infection, whereas EVT had a relatively higher incidence of pneumonia. Given the retrospective design, limited sample size, and potential residual confounding, these findings should be interpreted cautiously. Nevertheless, they provide preliminary evidence to inform clinical decisions and highlight the need for larger prospective studies.

## Data Availability

The raw data supporting the conclusions of this article will be made available by the authors, without undue reservation.
