# Supplementary material for: Comparison of endovascular and microsurgical treatment in patients with aneurysmal subarachnoid hemorrhage requiring external ventricular drainage
Source: Front Neurol. 2026 Jan 26;16:1708743. doi: 10.3389/fneur.2025.1708743 (PMC12884395; doi:10.3389/fneur.2025.1708743)

Table S1. Comparison of baseline characteristics between followed-up and lost-to-follow-up patients

|  | Follow-up  N=163 | Lost-to-Follow-up  N=34 | P Value |
| --- | --- | --- | --- |
| Age, y; mean (SD) | 63 (55-71) | 61 (52-66) |  |
| <65 | 106 (65.0) | 17 (50.0) | 0.100 |
| ≥65 | 57 (35.0) | 17 (50.0) |  |
| Sex, n (%) |  |  | 0.770 |
| Male | 57 (35.0) | 11 (32.4) |  |
| Female | 106 (65.0) | 23 (67.6) |  |
| Residence area, n (%) |  |  | 0.626 |
| Rural | 118 (72.4) | 26 (76.5) |  |
| Urban | 45 (27.6) | 8 (23.5) |  |
| Medical history, n (%) |  |  |  |
| Hypertension | 109 (66.9) | 22 (64.7) | 0.808 |
| Diabetes | 10 (6.1) | 3 (8.8) | 0.846 |
| Previous stroke | 25 (15.3) | 7 (20.6) | 0.450 |
| Lifestyle risk factors, n (%) |  |  |  |
| Smoking | 25 (15.3) | 5 (14.7) | 0.926 |
| Drinking | 17 (10.4) | 4 (11.8) | 1 |
| Presence of ICH or IVH, n (%) | 58 (35.6) | 14 (41.2) | 0.538 |
| HH grade, n (%) |  |  | 0.867 |
| I-III | 37 (50.7) | 9 (52.9) |  |
| IV-V | 36 (49.3) | 8 (47.1) |  |
| WFNS grade, n (%) |  |  | 0.252 |
| I-III | 88 (54.0) | 22 (64.7) |  |
| IV-V | 75 (46.0) | 12 (35.3) |  |
| Location of responsible aneurysm, n (%) |  |  | 0.711 |
| Anterior circulation arteries | 142 (87.1) | 31 (91.2) |  |
| Posterior circulation arteries | 21 (12.9) | 3 (8.8) |  |
| Size of the responsible aneurysm, mm |  |  | 0.394 |
| <5 | 85 (52.1) | 15 (44.1) |  |
| ≥5 | 78 (47.9) | 19 (55.9) |  |
| Multiple aneurysms, n (%) | 29 (17.8) | 5 (14.7) | 0.665 |
| Treatment |  |  | 0.606 |
| MST | 88 (54.0) | 20 (58.8) |  |
| EVT | 75 (46.0) | 14 (41.2) |  |
| Hydrocephalus before drainage | 15 (9.2) | 3 (8.8) | 1 |

Figure S1. The detailed mRS distribution.


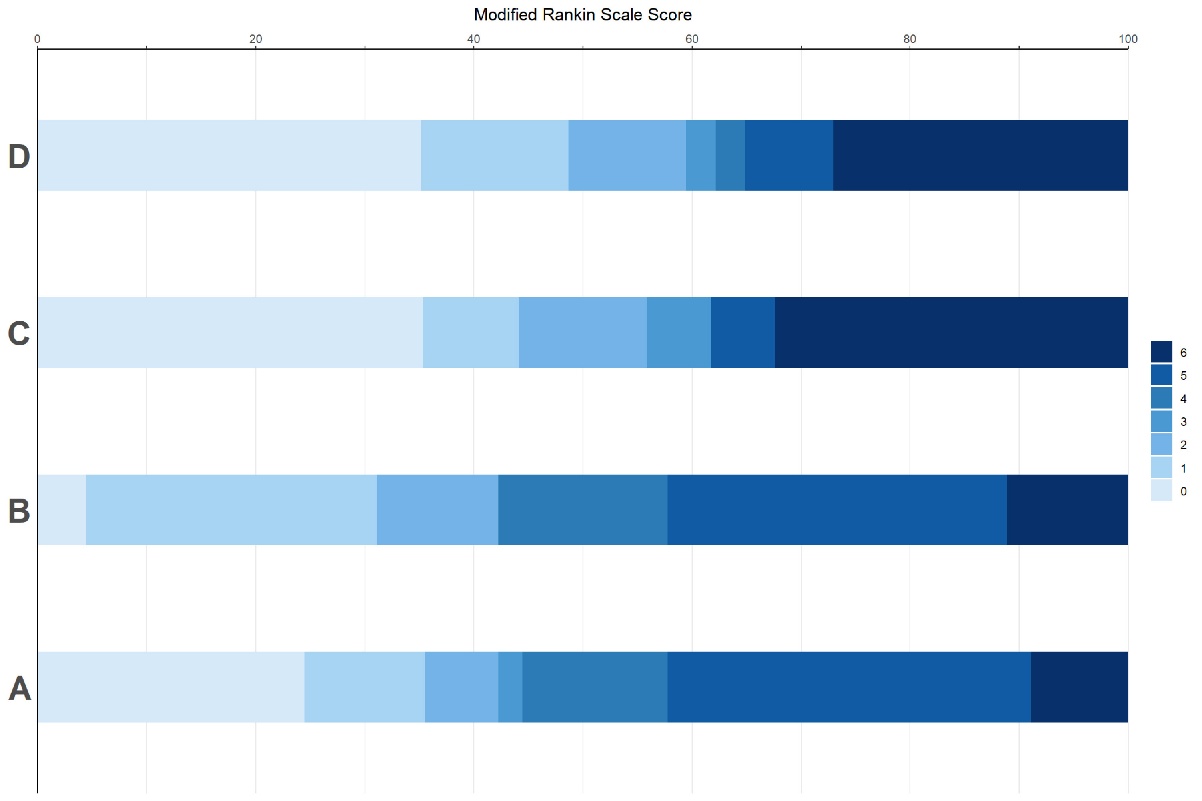

Supplement: Supplementary file 1 [file Table_1.DOCX]
